# Parents’ Perspectives on Self-Determination for Individuals with Intellectual Disabilities in Saudi Arabia

**DOI:** 10.3390/bs16020192

**Published:** 2026-01-29

**Authors:** Nujud A. Altuwayjiri, Nizar H. Bagadood, Mona F. Sulaimani

**Affiliations:** 1 Department of Special Education, Umm al-Qura University, Mecca 24382, Saudi Arabia; s44181294@st.uqu.edu.sa (N.A.A.); nbagadood@uqu.edu.sa (N.H.B.); 2 Department of Special Education, King Abdulaziz University, Jeddah 21589, Saudi Arabia

**Keywords:** self-determination, intellectual disability, parents

## Abstract

This study explores parents’ perspectives on self-determination for individuals with intellectual disabilities in the Saudi Arabian context. Using a qualitative approach, semi-structured interviews were conducted with six parents of individuals aged 15 to 34 years, and data were analyzed thematically. Findings reveal a clear tension between parents’ endorsement of self-determination as a valued goal and their emphasis on protection, safety, and family responsibility. Parents supported autonomy through structured choices, gradual skill-building, and daily routines, while restricting higher-stakes decisions due to concerns related to vulnerability and limited institutional and community support. These practices reflect culturally grounded caregiving responsibilities rather than resistance to self-determination. This study highlights the central role of families in shaping self-determination opportunities and underscores the influence of sociocultural values and systemic constraints. Implications emphasize the need for culturally responsive parent training, stronger school–family collaboration, and expanded community-based opportunities that support autonomy within safe environments.

## 1. Introduction

Saudi Arabia has experienced rapid social and economic development, accompanied by increasing attention to principles of equality, justice, and human rights. Central to these efforts is supporting individuals with disabilities to reach their full potential and participate actively in society. In recent years, greater emphasis has been placed on independence, employment, productivity, and personal choice. Saudi Arabia ratified the United Nations Convention on the Rights of Persons with Disabilities (CRPD) in 2008, which affirms the rights, autonomy, and social inclusion of persons with disabilities (Human Rights Commission, 2015; United Nations Human Rights, 2021). Article 19 of the Convention emphasizes the right to independent living, including the freedom to choose where and with whom to live. These commitments align with Saudi Vision 2030 and were further reinforced by the establishment of the Persons with Disabilities Authority in 2019 to promote empowerment and employment opportunities for individuals with disabilities (Al-Huwaiti, 2019).

National reports indicate that approximately 3.3% to 3.5% of Saudi youth aged 15–34 have disabilities, a population that faces significant challenges during critical life transitions. Navigating these transitions requires key self-determination skills, including independence, goal setting, self-advocacy, and decision-making (Hui & Tsang, 2012). Promoting self-determination is widely recognized as best practice for individuals with disabilities, particularly adolescents and adults with intellectual disabilities (Wehmeyer & Shogren, 2016). Research demonstrates that self-determination is associated with improved academic outcomes (Erickson et al., 2015; Chao & Chou, 2017), enhanced quality of life (Ismail, 2017; Chao, 2018), and successful transitions to adulthood, all of which support the broader objectives of Vision 2030. Consequently, self-determination has been integrated into evidence-based secondary education and transition programs (Test et al., 2016). Family involvement plays a critical role in this process by supporting decision-making, encouraging choice-making, and creating environments that respect individual abilities. However, these practices are shaped by cultural values, requiring professionals to adapt approaches to diverse social and familial contexts (Wu & Chu, 2012). Supporting self-determination also assists families in planning for their children’s futures and facilitating smoother transitions to adulthood (Wehmeyer, 2014).

As awareness of disability rights and social inclusion has increased, advocacy for self-determination among individuals with intellectual disabilities has gained prominence (Wehmeyer & Shogren, 2016). Self-determination is increasingly viewed as a vital and effective framework for enabling individuals to manage their lives and act autonomously (Al-Huwaiti, 2019). Despite its recognition as a fundamental human right under the CRPD, the implementation of self-determination remains challenging, particularly in contexts where guardians assume primary responsibility for decision-making (Skarstad, 2018). Even in settings with strong legal protections, individuals with disabilities often experience lower levels of self-determination than their peers, limiting opportunities for mobility, employment, and social participation (Chao & Chou, 2017). These challenges are frequently linked to restricted opportunities to practice self-determination in everyday life and to difficulties in communicating preferences and choices.

The concept of self-determination in the context of disability emerged in the early 1970s, with Swedish philosopher Bengt Nirje emphasizing respect for individual choice and independence. Later, Robert Perske introduced the notion of the “dignity of risk,” highlighting the importance of allowing individuals to make choices and learn through experience (Wehmeyer et al., 2017). During the 1980s, theoretical developments in motivation, particularly the work of Edward Deci and Richard Ryan, further shaped understandings of autonomy and self-regulation (Adams et al., 2017). By the late 1980s, self-determination had been incorporated into special education policy in the United States, emphasizing transition planning, social participation, and individualized programming. Over time, self-determination became central to educational and disability movements advocating for employment, independence, and inclusion, as well as the right of individuals with disabilities to lead meaningful lives free from discrimination (Brue & Wilmshurst, 2016; K. A. Shogren et al., 2017). Conceptually, self-determination encompasses recognizing personal desires, setting goals, engaging in self-advocacy, solving problems, and making informed decisions to achieve personal fulfillment (Calkins et al., 2011).

In recent decades, Saudi society has undergone a significant sociocultural transformation driven by modernization, globalization, and educational reform. These changes have contributed to the gradual emergence of values traditionally emphasized in Western contexts, such as autonomy, individual choice, and self-determination (Alrabiah, 2021). Although self-determination has historically been grounded in Western philosophical, psychological, and educational frameworks (K. A. Shogren & Wehmeyer, 2017), its relevance within Saudi society has become increasingly apparent as families, schools, and service systems navigate the balance between collective cultural values and growing recognition of individual rights and independence.

Understanding how self-determination is interpreted and practiced within the Saudi context—particularly within families of individuals with intellectual and developmental disabilities—is, therefore, critical for developing culturally responsive, evidence-based, and socially appropriate support systems. Within this evolving sociocultural landscape, the present study examines self-determination through the perspectives and experiences of families of individuals with intellectual disabilities in Saudi Arabia.

Despite the growing recognition of self-determination as a fundamental human right and an evidence-based practice for individuals with intellectual disabilities, limited attention has been given to how this concept is understood and enacted within Saudi families. Existing research has largely examined self-determination within Western contexts, while empirical studies addressing culturally specific family roles, values, and practices in Saudi Arabia remain scarce. Furthermore, many families continue to navigate tensions between protective caregiving, deeply rooted cultural norms, and emerging values related to autonomy and individual choice. This gap highlights the need for in-depth qualitative exploration of parental perspectives to better understand how families support, restrict, or shape opportunities for self-determination among individuals with intellectual disabilities in the Saudi context.

Although self-determination has been widely studied across educational and family contexts, much of the existing literature focuses on measurement, skill acquisition, or intervention outcomes, with less emphasis on the cultural meanings and familial dynamics that shape opportunities for self-determination. This concentration is particularly evident in family-focused research, where parents are positioned as primary gatekeepers of decision-making opportunities, especially in collectivist societies.

The purpose of this study is to explore parents’ perspectives on self-determination for individuals with intellectual disabilities in Saudi Arabia. Specifically, this study seeks to examine how families conceptualize self-determination, the strategies they use to foster autonomy and decision-making, and the challenges they encounter in balancing family values with the promotion of independence and self-determination in everyday life.

### 1.1. Research Questions

Based on the purpose of the study, the following research questions guided the investigation:

1. How do parents of individuals with intellectual disabilities in Saudi Arabia conceptualize self-determination?
2. What opportunities do families provide to support the development of self-determination skills among individuals with intellectual disabilities?
3. What challenges do parents perceive in fostering self-determination for their children within family, educational, and community contexts?

### 1.2. Significance of the Study

This study is significant as it contributes to the limited body of qualitative research on self-determination within Arab and Saudi contexts. By centering on the voices of parents, the study provides culturally grounded insights into the family’s role in shaping self-determination opportunities for individuals with intellectual disabilities. The findings are expected to inform educators, practitioners, and policymakers about culturally responsive practices that balance collective family values with the promotion of autonomy and individual rights. Moreover, the study responds directly to calls for contextualized research that moves beyond Western models and acknowledges the sociocultural transformations currently shaping Saudi society.

Research on the right to self-determination for individuals with intellectual disabilities has expanded across diverse cultural and educational contexts, with growing attention in Arab settings. Studies conducted in Arab countries have primarily relied on questionnaires and standardized scales to assess levels of self-determination among individuals with disabilities, their parents, and educators (Al-Ashram & Al-Shahawi, 2021; Al-Hassan, 2019; Al-Khatatbeh, 2018; Al-Shahili, 2022). This body of research reflects increasing awareness of self-determination as a critical developmental and educational outcome, while also revealing variability in opportunities and skill development across disability groups and educational systems.

Several studies have examined associations between self-determination and related psychosocial outcomes. For example, Ismail (2017) reported a positive relationship between self-determination skills and quality of life among adolescents with mild intellectual disabilities. Similarly, Al-Zboon and Smadi (2015) identified key indicators of self-determination within Jordan’s special education system, while Al-Shar’ah et al. (2018) highlighted variability in special education teachers’ understanding and implementation of self-determination skills. Collectively, these studies underscore the importance of contextual and systemic factors in shaping opportunities for self-determination.

Educators’ perspectives have also been widely explored. Research by Carter et al. (2011), Al-Quraini (2017), and McDonald (2018) consistently indicates strong endorsement of self-determination as an educational goal, alongside limited instructional implementation. Teachers and vocational assistants reported valuing core self-determination skills, such as choice-making and goal setting, yet cited constraints related to curriculum demands, time limitations, and insufficient training. These findings suggest a persistent gap between recognition of self-determination’s importance and its practical application within educational settings.

Family perspectives represent a particularly significant strand within the literature, given the central role of families in collectivist societies. Studies involving parents, individuals with disabilities, and educators have highlighted both opportunities and barriers to fostering self-determination. Al-Muaiqil and Al-Otaibi (2018) identified behavioral indicators of self-determination alongside systemic barriers, including the absence of structured curricula. Sagen and Ytterhus (2014) demonstrated that self-determination often occurs through informal, everyday decision-making, albeit with limited influence on major life choices.

Qualitative research has provided deeper insight into familial dynamics shaping self-determination. Studies by K. Shogren (2012), Chu (2018), Taylor et al. (2019), and Curryer et al. (2020) emphasized the complex balance families negotiate between autonomy and protection. Parents described supporting choice-making and independence while simultaneously expressing concerns related to safety, vulnerability, and future outcomes. These tensions were evident across age groups, from early childhood to adulthood, and were influenced by cultural expectations, family values, and perceived risks.

Contextual studies focusing specifically on parents further highlight these patterns. Carter et al. (2013), Villagomez (2016), and Alrabiah (2021) reported that parents generally value self-determination skills but often provide fewer opportunities for their children to practice them, particularly when disability severity, behavioral challenges, or communication difficulties are present. While these studies varied in methodology—ranging from large-scale questionnaires to qualitative interviews—they consistently positioned parents as key gatekeepers of self-determination opportunities.

Despite this growing body of literature, several gaps remain. First, empirical research in Arab contexts continues to rely heavily on quantitative approaches, with limited qualitative exploration of parents’ lived experiences. Second, few studies focus explicitly on adolescents and young adults with intellectual disabilities across transitional life stages. Finally, culturally grounded analyses that examine how family values, protection, and institutional constraints intersect to shape self-determination remain scarce. To address these gaps, the present study explores parents’ perspectives on self-determination for individuals with intellectual disabilities aged 15 to 34 within the Saudi Arabian context, using in-depth qualitative interviews to capture everyday practices, challenges, and opportunities.

## 2. Methodology

### 2.1. Research Paradigm and Methodological Approach

This study adopted a qualitative research approach in response to its central research question, which seeks an in-depth understanding of parents’ lived experiences and perspectives regarding self-determination for their children with intellectual disabilities. The research was guided by the phenomenological paradigm, which served as the overarching philosophical framework informing the study design.

Phenomenology focuses on exploring human experiences as they are lived and described by individuals, without imposing prior theoretical assumptions or external interpretations, with the aim of uncovering the essential meanings embedded within these experiences. Accordingly, this study employed descriptive phenomenology, which emphasizes rich, faithful descriptions of participants’ experiences as articulated in their own words rather than interpretive or explanatory theorizing.

This methodological approach was considered particularly appropriate, as it allows for the exploration of parents’ subjective meanings, everyday practices, and personal attitudes toward self-determination within their natural sociocultural context in Saudi Arabia.

### 2.2. Participants and Sampling

Participants included mothers and fathers of children with intellectual disabilities enrolled in special education or training programs in Saudi Arabia. Inclusion criteria required that participants meet the following points:

(1) Parents of a child with an intellectual disability;
(2) Have a child aged between 15 and 34 years, a critical developmental period for self-determination, aligned with the Saudi youth population;
(3) Be involved in educational or training programs linking school and home contexts;
(4) Reside in Saudi Arabia to ensure contextual relevance and accessibility.

Purposive sampling was used to recruit participants with direct and meaningful experience relevant to the study focus. Six participants meeting the criteria consented to participate and were scheduled for interviews.

### 2.3. Pilot Study

A pilot study was conducted prior to the main data collection to examine the clarity, relevance, and appropriateness of the interview guide and to identify potential challenges in the interview process. The pilot sample was purposively selected using the same inclusion criteria as the main study and consisted of two mothers of individuals with intellectual disabilities.

The first participant was a mother of a 16-year-old daughter with a moderate intellectual disability, and the second was a mother of a 17-year-old son with a mild intellectual disability. The pilot interviews lasted approximately 55 and 70 min, respectively, allowing participants sufficient time to express their views without fatigue.

Findings from the pilot study indicated the need to add clarifying questions, remove repetitive items, and reorganize sections of the interview guide. Technical challenges related to online interviewing platforms (e.g., Zoom) were also identified, leading to the adoption of a backup recording strategy using dual devices. These refinements strengthened the interview protocol and informed the procedures for the main study.

### 2.4. Data Collection Procedures

Semi-structured interviews were used as the primary method of data collection. Telephone interviews were selected to enhance participant comfort, ensure flexibility in scheduling, and reduce potential discomfort associated with face-to-face discussions of sensitive topics. This approach has been shown to facilitate openness and honest disclosure in qualitative research.

Participants were initially contacted via WhatsApp and provided with information about the study aims and procedures. Follow-up calls were conducted to explain the research in simple language, address questions, and obtain informed consent. All interviews were audio-recorded with participants’ permission, and confidentiality was assured through anonymization and secure data handling.

### 2.5. Data Analysis

Audio-recorded interviews were transcribed verbatim and analyzed using a systematic thematic analysis approach. Transcripts were read multiple times to achieve immersion in the data. Initial open coding was conducted to identify meaningful units related to parents’ perceptions, practices, and challenges surrounding self-determination.

Codes were compared across transcripts and grouped into broader categories. Through an iterative process of reviewing, refining, and collapsing categories, analytically coherent themes were developed. Constant comparison was employed throughout the analysis to ensure that themes reflect shared patterns across participants rather than isolated accounts.

### 2.6. Reflexivity and Trustworthiness

To ensure rigor and quality, the study adhered to the trustworthiness criteria proposed by Lincoln and Guba, including credibility, transferability, dependability, and confirmability.

Credibility was enhanced through audio-recording interviews, repeated engagement with the data, and member checking with selected participants to verify interpretive accuracy. Transferability was supported through rich descriptions of the study context, participants, and procedures. Dependability was ensured by systematic documentation of all research steps and external review of coding and thematic development. Confirmability was established through the use of direct quotations, transparent analytic documentation, and explicit links between data and interpretations.

Reflexivity was practiced throughout the research process, with the researchers engaging in ongoing reflection on their professional backgrounds, assumptions, and potential influence on data collection and interpretation. Reflexive notes and analytic discussions were used to minimize bias and ensure that participants’ voices remained central.

### 2.7. Data Saturation

Data collection continued until thematic saturation was achieved, at which point no new codes or themes emerged from participants’ accounts. Saturation was confirmed through iterative data collection and analysis, and additional interviews were deemed unnecessary once redundancy in themes was observed.

## 3. Results

This section presents the qualitative findings derived from the thematic analysis of parent interviews. Themes are reported with illustrative quotations (coded M.1–M.6) to remain close to participants’ perspectives.

### 3.1. Theme 1: Parents’ Awareness and Meanings of Self-Determination

This theme captures parents’ initial awareness of the concept of self-determination and how their understanding evolved through reflection on their children’s abilities and future needs. Across interviews, many parents described limited prior exposure to the term “self-determination” and reported a need for practical guidance on how to promote related skills at home and in school contexts. As one mother stated, “This is the first time I have heard of self-determination …” (M.3). Several parents linked this limited awareness to scarce local training opportunities and limited access to specialized resources.

Parents also emphasized that misunderstandings about disability rights may lower expectations regarding their children’s ability to make choices, self-advocate, or participate in decisions. Some parents indicated that they had previously assumed these skills were primarily relevant to non-disabled individuals. After discussing the concept, participants generally expressed stronger endorsement of self-determination as a meaningful goal for their children.

### 3.2. Theme 2: Supported Choice-Making Within Family Values and Protection Priorities

This theme illustrates how parents frame self-determination as a supported and bounded process, in which choice-making is permitted within limits shaped by safety concerns, family values, and protective responsibilities. Parents commonly described providing opportunities for “simple” choices (e.g., food, clothing options, leisure activities) while retaining control over decisions perceived as higher stakes. For example, one parent explained, “He chooses from the menu… just simple things” (M.3), whereas another reported that most decisions were made by family members due to concerns about decision-making capacity (M.2).

Participants frequently framed restrictions on choice-making as protective practices linked to safety, health, and social/religious norms. Some parents described offering only pre-screened options aligned with family values (e.g., dress choices) to avoid outcomes they viewed as inappropriate (M.6). Others described limiting independent community participation due to fears of exploitation and limited self-defense skills.

### 3.3. Theme 3: Promoting Autonomy and Problem-Solving in Daily Life

This theme highlights parents’ everyday practices aimed at fostering autonomy and problem-solving through gradual support withdrawal and structured opportunities within daily routines. Despite protective boundaries, parents described multiple home-based strategies to strengthen autonomy and problem-solving. A common approach involved gradually withdrawing support and encouraging repeated attempts during self-care and household routines (e.g., hygiene, preparing simple foods). One parent noted, “I tell her, ‘No, you brush yourself,’ so she learns on her own and tries multiple times” (M.6).

Parents also described using reminders (visual schedules or verbal prompts) to support routine management and independence over time. In addition, several parents reported leveraging personal interests and preferred rewards to increase engagement and persistence (e.g., completing tasks followed by a favored item or activity).

### 3.4. Theme 4: Limited Social and Educational Opportunities and Institutional Barriers

This theme reflects parents’ perceptions of external structural and institutional barriers that constrain opportunities for self-determination beyond the home environment. Beyond the home, parents emphasized constraints in community and educational environments that reduce opportunities for self-determination. Parents described limited access to diverse training, employment, recreation, and structured social participation opportunities—often requiring constant accompaniment, which was not always feasible for families (e.g., gym participation requiring a supervisor).

In school contexts, parents highlighted weak school–family partnerships, limited individualized planning, and curricula that prioritize academic content while providing fewer structured opportunities for choice-making, goal-setting, or participation in extracurricular activities. In contrast, several parents described more supportive practices in specialized centers, including more individualized planning, stronger communication with families, and broader activity-based learning opportunities.

## 4. Discussion

This study provides an in-depth examination of parents’ perspectives on self-determination for individuals with intellectual disabilities within the Saudi Arabian context. The findings reveal a consistent and nuanced tension between parents’ endorsement of autonomy and their simultaneous prioritization of protection. Across participants’ accounts, parents strongly valued self-determination as an essential goal for their children’s future independence; however, they often restricted higher-stakes decisions due to concerns related to safety, vulnerability, social accountability, and family responsibility. This tension reflects a broader pattern documented in international literature, where families—particularly in collectivist sociocultural contexts—frequently function as primary gatekeepers of decision-making opportunities for individuals with intellectual disabilities (K. Shogren, 2012; Saaltink et al., 2012; Curryer et al., 2020; Bigby et al., 2022).

Importantly, the findings suggest that parental protection should not be interpreted as resistance to self-determination or as a deficit-oriented practice. Rather, protective decision-making emerges as a culturally grounded caregiving responsibility shaped by family norms, moral obligations, religious values, and contextual risks. Within the Saudi context, parents’ heightened sense of responsibility is further influenced by structural constraints, including limited inclusive community programs, institutional requirements for constant accompaniment, restricted employment and recreational opportunities, and inconsistent school–family collaboration. Under these conditions, parents’ cautious regulation of autonomy can be understood as an adaptive strategy aimed at safeguarding their children’s well-being rather than denying their rights.

At the same time, parents reported actively fostering autonomy within carefully defined and socially acceptable boundaries. Participants described a range of everyday practices that supported self-determination, including offering structured and limited choices, gradually withdrawing support as skills developed, encouraging retries and problem-solving, and embedding autonomy-building opportunities within daily routines. These practices align with prior research emphasizing supported decision-making and scaffolded autonomy as key mechanisms for developing self-determination, particularly for individuals with intellectual disabilities (Chu, 2018; Taylor et al., 2019; Curryer et al., 2020). Rather than conceptualizing self-determination as an all-or-nothing capacity, parents viewed it as a set of competencies that can be incrementally strengthened through intentional family practices and enabling environments.

These findings reinforce contemporary conceptualizations of self-determination as a relational and context-dependent process rather than an exclusively individual attribute. As articulated by K. A. Shogren and Wehmeyer (2017), self-determination develops through opportunities shaped by social relationships, cultural expectations, and systemic structures. In the present study, parents’ efforts to balance autonomy and protection illustrate how self-determination is continuously negotiated within family systems, where cultural values, safety considerations, and institutional limitations intersect. Recognizing this complexity is essential to avoid deficit-based interpretations of parental involvement and to design supports that are both culturally responsive and practically feasible.

Beyond confirming existing literature on family involvement in self-determination, the present study offers a culturally grounded contribution by reframing parental protection not as an obstacle, but as an adaptive and relational strategy shaped by sociocultural and systemic conditions. The findings suggest that self-determination in collectivist contexts may be enacted through supported and negotiated autonomy rather than independent decision-making. This perspective extends prevailing self-determination frameworks, which have largely been developed within Western individualistic paradigms, by highlighting culturally responsive pathways to autonomy support.

Building on these findings, several implications for practice emerge. First, accessible and culturally sensitive parent training programs are needed to enhance understanding of self-determination, supported decision-making, and graduated autonomy-building strategies. Such programs should acknowledge parents’ protective roles while equipping them with practical tools to expand safe opportunities for choice-making and participation. Second, strengthening school–family collaboration is critical. Shared goal-setting, transparent communication, and coordinated transition planning can help align home and school efforts and ensure that self-determination is intentionally supported across settings. Finally, expanding community-based programs that offer supervised yet autonomy-promoting opportunities—such as vocational training, recreational activities, and social participation initiatives—can reduce families’ perceived risks and create environments in which individuals with intellectual disabilities can practice self-determination in meaningful and socially supported ways.

### Limitations and Future Directions

As a qualitative study, the findings reflect the experiences and perspectives of the participating parents and are not intended to be statistically generalized. Future research could strengthen the evidence base by triangulating parent perspectives with those of individuals with intellectual disabilities, educators, and service providers. Longitudinal studies may also examine how opportunities for self-determination evolve across developmental stages and transitions, particularly from adolescence to adulthood. Additionally, further research is needed to explore gendered differences in opportunities and expectations, as well as the impact of emerging inclusive policies and community initiatives within the Saudi context.

## 5. Conclusions

The findings of this study highlight the potential value of culturally responsive family support and educational practices that encourage self-determination within socially embedded contexts. While the results are based on a small qualitative sample, they point to promising directions for future research and practice that may be further examined through larger-scale and mixed-methods studies.

This study contributes to the literature by illuminating how parents in Saudi Arabia understand and support self-determination for individuals with intellectual disabilities within a complex interplay of cultural values, protective responsibilities, and systemic constraints. The findings demonstrate that parents value autonomy and independence for their children but often regulate decision-making opportunities in response to safety concerns, social expectations, and limited institutional support. These practices do not reflect opposition to self-determination, but rather culturally grounded caregiving strategies shaped by contextual realities.

Supporting the right to self-determination for individuals with intellectual disabilities is a shared responsibility that extends across family, educational, and community systems. Families play a foundational role by fostering daily living skills, gradual independence, and supported decision-making within the home. Schools serve as critical gateways for expanding opportunities beyond the family by promoting inclusive practices, collaborative planning, and meaningful participation in academic and non-academic activities. At the community level, accessible employment, training, healthcare, and psychosocial support are essential for translating self-determination from a conceptual ideal into lived experience.

Ultimately, meaningful progress toward self-determination requires coordinated, culturally responsive efforts that respect family values while expanding safe and structured opportunities for autonomy. By aligning family practices, educational support, and community resources, stakeholders can better support individuals with intellectual disabilities in developing the skills, confidence, and agency needed to lead self-directed and fulfilling lives.

## Data Availability

The data presented in this study are not publicly available due to ethical and privacy considerations related to qualitative interview data. Data may be available from the corresponding author upon reasonable request.
